# Cysteine-rich protein 2 accelerates actin filament cluster formation

**DOI:** 10.1371/journal.pone.0183085

**Published:** 2017-08-16

**Authors:** Takanori Kihara, Yasunobu Sugimoto, Satoko Shinohara, Shunpei Takaoka, Jun Miyake

**Affiliations:** 1 Department of Life and Environment Engineering, Faculty of Environmental Engineering, The University of Kitakyushu, Hibikino, Wakamatsu, Kitakyushu, Fukuoka, Japan; 2 Department of Biotechnology, Graduate School of Engineering, Nagoya University, Furo-cho, Chikusa-ku, Nagoya, Aichi, Japan; 3 Department of Mechanical Science and Bioengineering, Graduate School of Engineering Science, Osaka University, Machikaneyama, Toyonaka, Osaka, Japan; Osaka Shiritsu Daigaku, JAPAN

## Abstract

Filamentous actin (F-actin) forms many types of structures and dynamically regulates cell morphology and movement, and plays a mechanosensory role for extracellular stimuli. In this study, we determined that the smooth muscle-related transcription factor, cysteine-rich protein 2 (CRP2), regulates the supramolecular networks of F-actin. The structures of CRP2 and F-actin in solution were analyzed by small-angle X-ray solution scattering (SAXS). The general shape of CRP2 was partially unfolded and relatively ellipsoidal in structure, and the apparent cross sectional radius of gyration (*R*_*c*_) was about 15.8 Å. The predicted shape, derived by *ab initio* modeling, consisted of roughly four tandem clusters: LIM domains were likely at both ends with the middle clusters being an unfolded linker region. From the SAXS analysis, the *R*_*c*_ of F-actin was about 26.7 Å, and it was independent of CRP2 addition. On the other hand, in the low angle region of the CRP2-bound F-actin scattering, the intensities showed upward curvature with the addition of CRP2, which indicates increasing branching of F-actin following CRP2 binding. From biochemical analysis, the actin filaments were augmented and clustered by the addition of CRP2. This F-actin clustering activity of CRP2 was cooperative with α-actinin. Thus, binding of CRP2 to F-actin accelerates actin polymerization and F-actin cluster formation.

## Introduction

Cysteine-rich proteins (CRPs; also referred to as cysteine and glycine-rich proteins; CSRPs) are muscle cell differentiation related proteins [1–7] and they consist of two LIM domains, which are double zinc-finger-like structures that mediate protein-protein interactions, and two glycine-rich regions. CRP family members (CRP1, CRP2/SmLIM, and CRP3/MLP) share a highly sequence homology, but their expression patterns differ depending on the type of tissue [8]. For instance, CRP1 is expressed in multiple smooth muscle organs and CRP2 is expressed in vascular smooth muscle cells (SMCs) [9–11], and these proteins act as cofactors for SMC differentiation [4]. In proepicardial cells, CRP2 forms complex with serum response factor (SRF) and GATA proteins in the nucleus, and this complex strongly activates SMC-specific gene expressions [4]. Furthermore, CRP2 acts as a transcriptional co-adaptor remodeling silent SMC gene chromatin [12,13]. As differentiation of proepicardial cells into SMCs, CRP2 translocates to the actin cytoskeleton [4]. CRP2 prefers to associate with actin filaments [6,14], and the translocation is entirely regulated by the formation of actin stress fibers in conjunction with SMC differentiation [6]. Therefore, it is thought that CRP2 plays different roles in these different locations, with the cytoplasmic CRP2 being involved in the assembly and maintenance of the actin cytoskeleton [7].

Numerous cellular structures, such as lamellipodia, filopodia, and muscle filaments are constructed using the actin cytoskeleton. The actin cytoskeleton consists of filamentous actin (F-actin), which is a helical assembly of globular actin (G-actin) and numerous actin-binding proteins. The F-actin is a flexible, ribbon-like filament with a diameter of 100 Å [15]. A large number of actin binding proteins have been shown to participate in the assembly and disassembly of actin molecules [16]. Furthermore, parallel assembly of F-actin filaments by bundling proteins forms F-actin bundles, and cross assembly of F-actin by crosslinking proteins forms a network structure of F-actin. The bundling of F-actin is generally used for mechanical support of cells and the F-actin network supports the plasma membrane and is involved in cell shape, motility, and adhesion [17,18]. Thus, the role of actin binding proteins in actin dynamics, structure, and assembly is important for cell life.

CRP2 strongly associates with F-actin in comparison to G-actin [6] and promotes actin bundling [19]. In this study, we examined the structural effects of CRP2 on F-actin. Particularly, we examined the molecular structure of CRP2 in solution and the supramolecular structure of F-actin interacting with CRP2 by small angle X-ray solution scattering (SAXS). The cross-sectional radius of gyration (*R*_*c*_) of the actin-based filaments was nearly constant even with the addition of CRP2. However, CRP2 did accelerate the filament formation and supramolecular clustering of F-actin, and this activity of CRP2 was cooperative with α-actinin. This study provides additional information about the interaction of CRP2 and actin filaments.

## Materials and methods

### Materials

Recombinant mouse CRP2 protein, N-terminal LIM domain and glycine-rich region (N-LIM) of CRP2 and C-terminal LIM domain and glycine-rich region (C-LIM) of CRP2 were prepared as described previously [6]. Purified and quantified these proteins were analyzed by SDS-PAGE. We used total more than 4 batches of recombinant CRP2 protein and 1 batch of each N-LIM and C-LIM protein in this study. Actin protein (from Bovine skeletal muscle) was purchased from Sigma-Aldrich (St. Louis, MO). Actin was firstly solved with G-buffer (5 mM Tris-HCl, pH 8.0, 0.2 mM CaCl_2_, 0.2 mM ATP, and 0.5 mM DTT). After the actin solution was centrifuged at 20,000 g for 30 min at 4°C, we collected supernatant of the solution and used for experiments as G-actin. We used several batches of actin protein in this study. Alexa Fluor 488 (Alexa488)-labeled actin was purchased from Life Technologies Japan Ltd. (Tokyo, Japan). Actin protein containing 10% of pyrene-labeled actin was purchased from Hypermol (Bielefeld, Germany). α-actinin was purchased from Cytoskeleton Inc. (Denver, CO). Polyethylene glycol (20 k) was purchased from Nakalai tesque (Kyoto, Japan). Other reagents were purchased from Wako Pure Chemical Industries Ltd. (Osaka, Japan), Life Technologies Japan Ltd. (Tokyo, Japan), Sigma-Aldrich, or Takara Bio Inc (Shiga, Japan).

### SAXS experiments

SAXS measurements were performed using a monochromatized synchrotron X-ray beam (wavelength, 1.50 Å). Synchrotron radiation from a bending magnet source in the electron storage ring at the Photon Factory (KEK, Tsukuba, Japan), operated at 2.5 GeV with a ring current between 350 and 450 mA, was selected and collimated with the double focusing optics installed at beamline 6A. In order to allow small-angle measurements required for the present experiments, we used a beam of dimensions 0.20 mm (vertical) × 0.20 mm (horizontal) at the detector plane with a specimen-to-detector distance of 2280 mm. Scattered X-ray were recorded by a XRII-CCD detector (C7300) (Hamamatsu Photonics K.K., Shizuoka, Japan). The beam intensity incident at the specimen was monitored with an ion chamber placed in front of the specimen. The sample was inserted in a temperature-controlled cell with two mica windows, through which X-ray passed, and the temperature was maintained at 25°C. Normally, 12–18 measurements for each prepared sample were taken at different concentrations. Each exposure time of X-ray was 10 s to minimize radiation damage of the sample. Buffer scattering measurements were performed periodically throughout the measurement sequence. Each intensity image was integrated in the circumferential direction. Then, after correction for variations in the beam intensity, as measured by the current values from the ion chamber, we obtained net intensity data, *I(Q*,*c)*, by subtracting the buffer scattering from the sample solution scattering, where *Q* is the momentum transfer vector (= *4πsinθ/λ*, in which *2θ* and *λ* are the scattering angle and the wavelength of the monochromatized X-ray used, respectively) and *c* is the protein concentration.

For CRP2 measurements, CRP2 solutions were prepared at 4.3, 5.7, 7.1, 8.6, and 11 mg/mL, in phosphate-buffered saline with 0.05% Tween 80. For F-actin measurements, F-actin was formed by incubating actin protein (0.40 and 0.80 mg/mL) with F-buffer (5 mM Tris-HCl, pH 8.0, 100 mM KCl, 2 mM MgCl_2_, 0.2 mM CaCl_2_, 1 mM ATP, 0.5 mM DTT) and 3.0-fold amount of phalloidin at 37°C for 120 min. F-actin-CRP2 samples were prepared by mixing the F-actin solution (final concentration of 0.65 mg/mL) and each CRP2 solution (final concentration of 0.55, 1.1, and 1.65 mg/mL) and further incubating this mixture at 37°C for 60 min.

### Analysis of SAXS intensity data

First, we calculated the average intensity data, $\bar{I(Q,c)}$, from the 12–18 measured net intensity data, *I(Q*,*c)*, for each protein concentration. The Kratky plot, *I(Q*,*c)·Q*^*2*^ vs. *Q* plot [20], was used for each $\bar{I(Q,c)}/c$ data. The apparent cross-sectional radius of gyration, *R*_*c*_*(c)*, was determined from the slope of the modified Guinier plot for a rod-like particle (ln[*I(Q*,*c)·Q*] vs. *Q*^*2*^) [20], in which we used $\bar{I(Q,c)}/c$, in the narrow *Q* range (up to *Q = 0*.*78/R*_*c*_). The slope of the Guinier plot (ln[*I(Q*,*c)·Q*] vs. *Q*^*2*^) shows $-\frac{R_{c(c)}^{2}}{2}$. The final *R*_*c*_ value at *c = 0* was determined by means of a least-squares fitting to the data points at various protein concentrations.

The pair-distance distribution function, *p(r)*, was calculated by the program GNOM [21] using the merged data of low concentrated and high concentrated $\bar{I(Q,c)}/c$ data; in the narrow *Q* range (*Q ≤* 0.04834), we used low concentrated $\bar{I(Q,c)}/c$ data, and in the wide *Q* range (*Q >* 0.04834), we used high concentrated $\bar{I(Q,c)}/c$ data. The maximum chord length, *D*_*max*_, of the molecule was determined according to the total estimation value of the GNOM program [21]. The program DAMMIF [22] was used to construct three-dimensional molecular envelopes that fit to the merged $\bar{I(Q,c)}/c$ data. The program was run 10 times independently and the obtained models were sorted and averaged by the DAMAVER program [23]. Finally, we refined the averaged model by the DAMMIN program [24].

For the F-actin analysis, we obtained net intensity data, *I(Q*,*c)*, by subtracting the buffer and CRP2 scatterings from the sample solution scattering, where *c* is the actin concentration. We calculated the average intensity data, $\bar{I(Q,c)}$, from the 12–18 measured net intensity data, *I(Q*,*c)*, in each actin protein concentration. The apparent cross-sectional radius of gyration, *R*_*c*_, was determined from the slope of the modified Guinier plot for rod-like particles (ln[*I(Q*,*c)·Q*] vs. *Q*^*2*^) [20], in which we used $\bar{I(Q,c)}/c$, in the narrow *Q* range (up to *Q = 1*.*7/R*_*c*_). The slope of the modified Guinier plot (ln[*I(Q*,*c)·Q]* vs. *Q*^*2*^) shows $-\frac{R_{c}^{2}}{2}$.

The SAXS analyzed raw data is available in supplementary S1 File.

### Confocal laser scanning microscopy

F-actin was formed by incubating 0.042 mg/mL of actin protein (an equivalent mixture of Alexa Fluor 488-labeled actin and non-labeled actin) with F-buffer containing 8% PEG and various concentrated CRP2 at 23°C for 4 h. The solution was spread onto the slide glass and covered with cover glass. The Alexa Fluor-labeled F-actin was observed by confocal laser scanning microscopy (Nikon C2, Nikon, Tokyo, Japan). For analyze single bundled F-actin length, F-actin was formed by incubating 0.17 mg/mL of actin protein (an equivalent mixture of Alexa Fluor 488-labeled actin and non-labeled actin) with F-buffer containing 8% PEG and 0.022 mg/mL of CRP2 at 23°C for 4 h. The Alexa Fluor-labeled F-actin was observed by confocal laser scanning microscopy. From the obtained fluorescence images, contour length of single bundled F-actin were measured using Image J software (NIH, Bethesda, MD). The filament lengths for each group were compared by analysis of variance followed by Mann-Whitney U test. For the experiments in presence of α-actinin, F-actin was formed by incubating 0.042 mg/mL of actin protein (an equivalent mixture of Alexa Fluor 488-labeled actin and non-labeled actin) with F-buffer containing 8% PEG, with or without 0.022 mg/mL CRP2 and 0.01 mg/mL α-actinin at 23°C for 2.5 h.

### Actin polymerization assay

Actin monomers (0.4 mg/mL) containing 10% of pyrene labeled in G-buffer were induced to polymerize by addition of a 1/10th volume of 10 × F-buffer (5 mM Tris-HCl, pH 8.0, 1.0 M KCl, 20 mM MgCl_2_, 0.2 mM CaCl_2_, 10 mM ATP, and 0.5 mM DTT) in the absence or in the presence of CRP2 (0.026, 0.11, 0.22 mg/mL). The solution was mixed and transferred into quartz micro-cell, and then the fluorescence of the pyrene (excitation: 365nm; emission: 407 nm) was recorded using a F4500 spectrofluorometer (Hitachi, Tokyo, Japan) at 23°C. The time from mixing to starting to measure was fixed at 90 sec.

### Low-speed actin co-sedimentation assay

0.17 mg/mL of actin protein was copolymerized with 0.088 mg/mL of CRP2, 0.04 mg/mL of N-LIM, and 0.04 mg/mL of C-LIM for 2 h at 23°C in F-buffer containing 8% PEG. After polymerization, the solution was centrifuged at 20,000 × g for 30 min at 23°C. The resulting pellets were analyzed by SDS-PAGE and silver staining (2D Silver Stain-II, Cosmo Bio, Tokyo, Japan).

## Results

### SAXS analysis of CRP2

**Fig 1:**
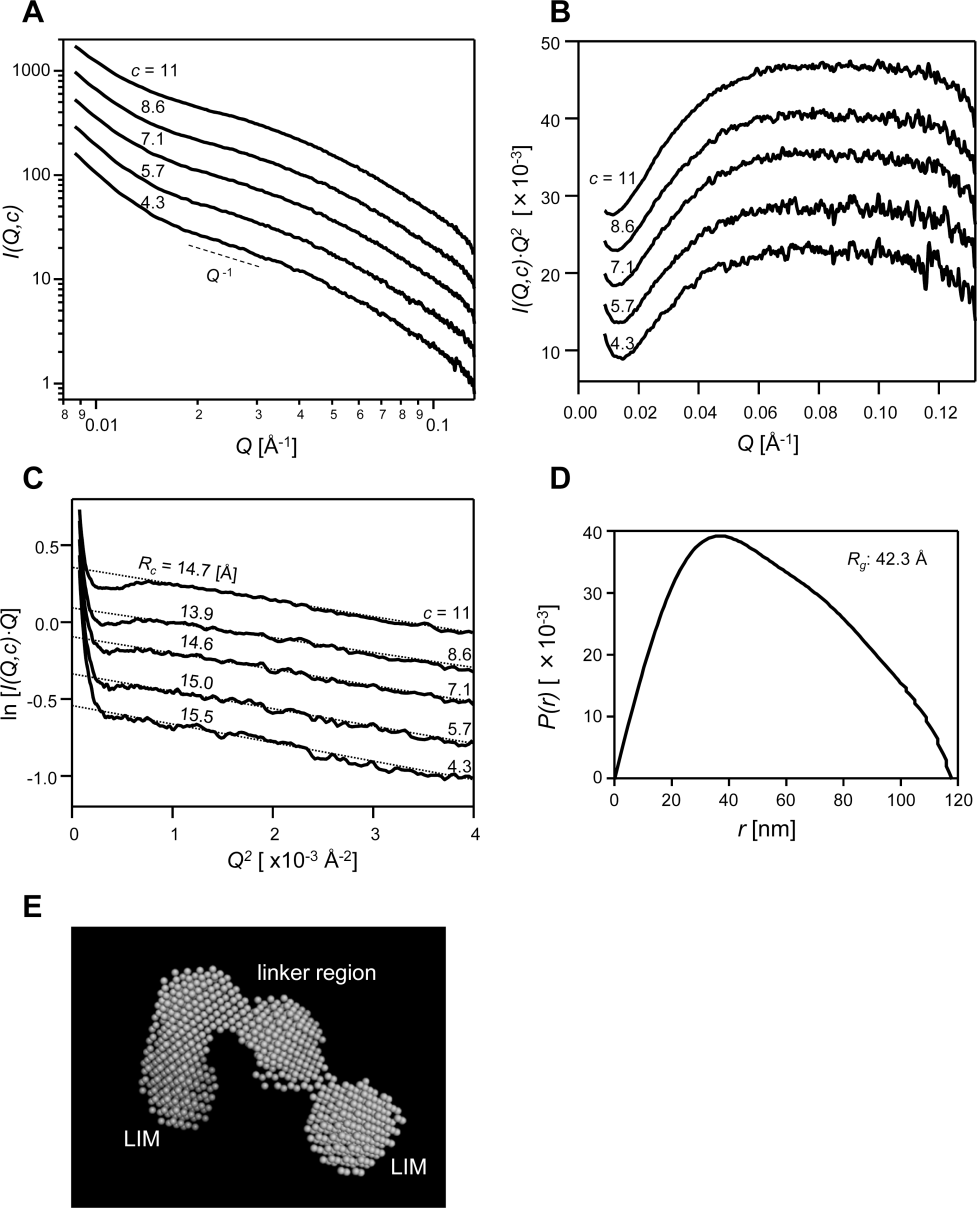
SAXS curves and predicted shape of CRP2 in solution. (A) The SAXS intensity data (*I(Q*,*c)*) for each CRP2 concentration. The CRP2 concentration, *c* (mg/mL), is shown in each curve. The used scattered data *I(Q*,*c)* is $\bar{I(Q,c)}/c$. For clarity, the curves are shifted by an arbitrary unit on the *I(Q*,*c)* axis. The dashed line shows the slope of *Q*^*-1*^. (B) The Kratky Plot (*I(Q*,*c)·Q*^*2*^–*Q*) of the scattered data for each CRP2 concentration in solution. The CRP2 concentration, *c* (mg/mL), is shown in each curve. The used scattered data *I(Q*,*c)* is $\bar{I(Q,c)}/c$. For clarity, the curves are shifted by an arbitrary unit on the *I(Q*,*c)·Q*^*2*^ axis. (C) The modified Guinier Plot for rod objects (ln[*I(Q*,*c)·Q*]–*Q*^*2*^) using the scattered data for each CRP2 concentration. The used scattered data is $\bar{I(Q,c)}/c$. The CRP2 concentration, *c* (mg/mL), is shown in the right part of each curve. The dashed lines represent extrapolated modified Guinier fits. The calculated *R*_*c*_ value from the fitting is shown beside each line. For clarity, the curves are shifted by an arbitrary unit on the ln[*I(Q*,*c)·Q*] axis. (D) The pair-distance distribution function, *p(r)*, curve of CRP2. The maximum chord length, *D*_*max*_, is 118 Å. (E) The general shape of CRP2 in solution was derived from the *ab initio* shape prediction programs, DAMMIF and DAMMIN. The maximum length of this shape was 118 Å. Estimated LIM domains and linker part are shown. The PDB file of CRP2 dummy model is available in supplementary S2 File.

The structure of quail CRP2 in solution was determined by NMR [25]. The two LIM domains of CRP2 are structurally and dynamically independent from each other. To understand the structural interaction between CRP2 and actin filaments in solution, we first determined the structural characteristics of CRP2 in solution by SAXS analysis. In this study, we used recombinant mouse CRP2 with (His)_6_ tag at the C-terminus, which was expressed in *E*. *coli* and purified for determining the interaction of CRP2 with F-actin [6]. Fig 1A shows the SAXS data, $\bar{I(Q,c)}$, of each concentrated CRP2 solution. The $\bar{I(Q,c)}$ in the small-angle region (Q ≤ 1.7 × 10^−2^ Å^-1^) showed an upturn, which indicates some aggregation of the molecules in solution. The concentration of the CRP2 in this study was 0.20–0.50 mM, and these high concentrations of CRP2 slightly aggregated in solution.

First, we checked the structural fold of CRP2 using Kratky plot (*I(Q*,*c)·Q*^*2*^ vs. *Q* plot), where *I(Q*,*c)* is the $\bar{I(Q,c)}/c$ and *Q* is the momentum transfer vector (4πsinθ/λ). In the case of a well-folded globular protein, the Kratky plot will exhibit a “bell-shaped” peak at low *Q* and converge to the *Q* axis at high *Q*, while for an unfolded protein, the plot will lack the peak and slightly increase at high *Q* [20]. The Kratky plot of CRP2 showed gradual peak that was slightly decreased at high *Q* (Fig 1B). Thus, CRP2 appears to be partially unfolded in solution.

The decreased ratio of SAXS intensity for *Q* provides the general shape of the materials. The *I(Q)* of CRP2 roughly correlated with *Q*^*-1*^, indicating that CRP2 was a rod or ellipsoidal object. Furthermore, the ellipsoidal particle, whose ratio of diameter to length was more than 2, shows the Guinier linearity of the cross section curves [20]. We then checked the Guinier linearity of CRP2 in the cross sectional modified Guinier plot (ln[*I(Q*,*c)·Q*] vs. *Q*^*2*^ plot) for each CRP2 concentration (Fig 1C). In the region of 7.5 × 10^−4^ ≤ Q^2^ Å^-2^, the intensities decreased linearly, and in the smaller angle region, 4.0 × 10^−4^ ≤ Q^2^ ≤ 7.5 × 10^−4^ Å^-2^, the intensities showed lower values than the values of the extended lines (Fig 1C). This trend of the cross sectional plot is observed in ellipsoidal particles [20]. Thus, the structural character of CRP2 was hypothesized to be ellipsoidal. The apparent radii of cross sectional gyration, *R*_*c*_*(c)*, was then calculated from the slopes of the lines in Fig 1C. The fitting range of a straight line for the modified Guinier plot was 0.9 × 10^−3^ Å^-2^ ≤ Q^2^ ≤ 2.5 × 10^−3^ Å^-2^, which is 0.4 ≤ *Q·R*_*c*_ ≤ 0.8. The apparent *R*_*c*_ value, extrapolated to *c = 0*, was 15.9 (0.74) Å.

We then merged low concentrated and high concentrated $\bar{I(Q,c)}/c$ data to predict the shape of CRP2. Using the merged data, the pair-distance distribution function, *p(r)*, was calculated by the program GNOM (Fig 1D) [21]. The maximum chord length, *D*_*max*_, of the molecule was determined to be 118 Å according to the total estimation value of GNOM program. The CRP2 shape was then modeled with the use of the *ab initio* shape prediction program DANMMIF [22] from the merged data, and the *D*_*max*_, 118 Å. The obtained 10 dummy models were averaged by the program DAMAVER [23] and then the final *ab initio* model was refined by the DAMMIN program [24]. Fig 1E shows the culminating dummy model of CRP2 in solution. The CRP2 model contains roughly four tandem cluster structures. The NMR-determined structure of the full-length quail CRP2 shows that the folded LIM domains are connected via a structurally disordered flexible linker [25]. Furthermore, our Kratky plot of CRP2 showed partial unfolded structure (Fig 1B). Thus, both end clusters in our model indicate folded LIM domains and the middle clusters show a flexible linker of LIM domains (Fig 1E).

### SAXS analysis of CRP2 bound F-actin

**Fig 2:**
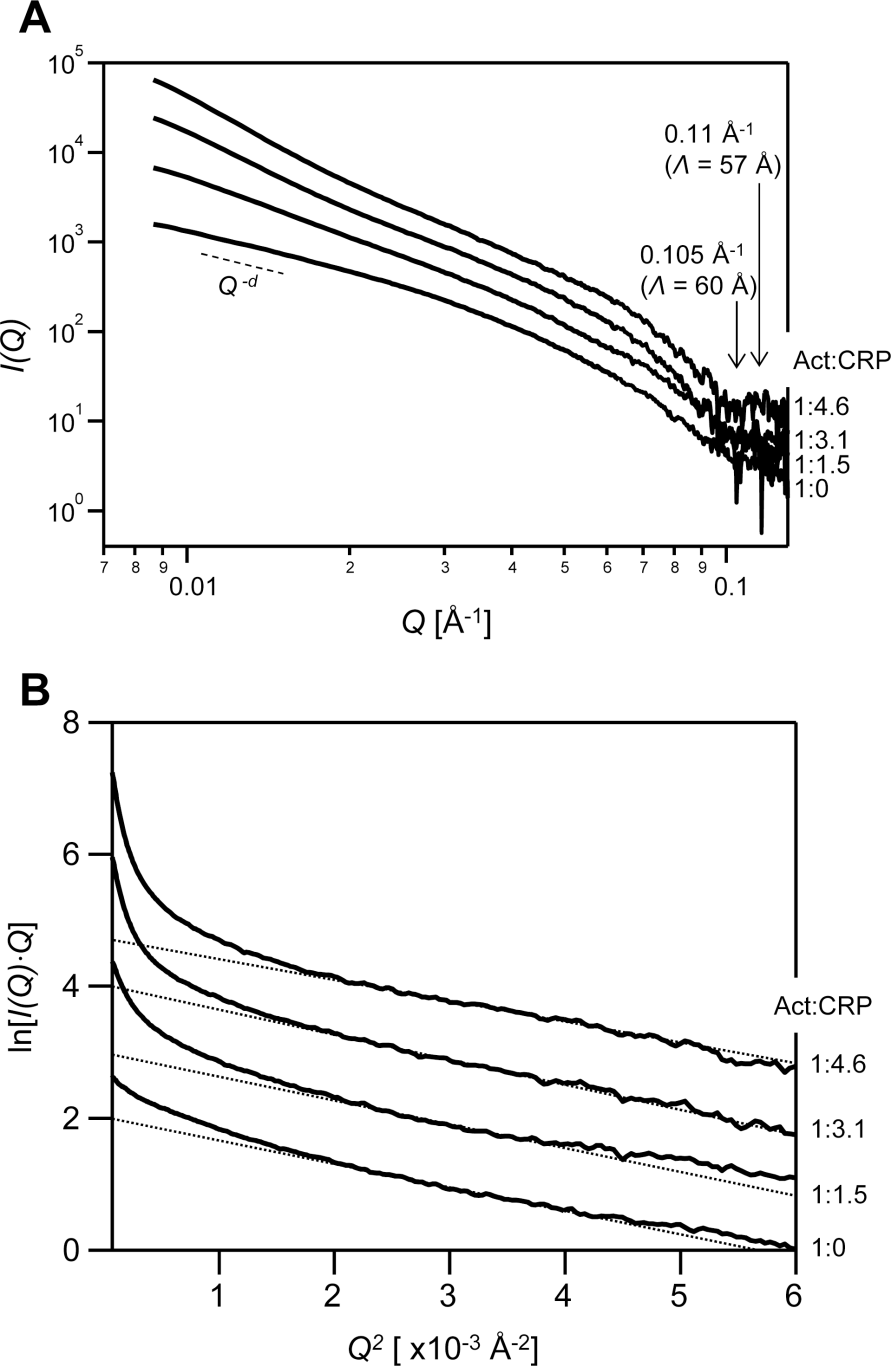
SAXS curves for F-actin in solution. (A) The SAXS intensity data (*I(Q)*) of F-actin with or without CRP2. The ratio of F-actin and CRP2 is shown on the right part of each curve. The used scattered data *I(Q)* is $\bar{I(Q,c)}/c$, where *c* is the concentration of actin. For clarity, the curves are shifted by an arbitrary unit on the *I(Q)* axis. The dashed line shows slope of scattered data (*Q*^*-d*^). The scattered valley and peak are indicated with arrows. *Λ* is the Bragg distance of each position. (B) The modified Guinier Plot (ln[*I(Q)·Q*]–*Q*^*2*^) using the scattered data for each F-actin solution. The used scattered data is $\bar{I(Q,c)}/c$. The ratio of F-actin and CRP2 is shown on the right part of each curve. The dashed lines represent extrapolated Guinier fits. For clarity, the curves are shifted by an arbitrary unit on the ln[*I(Q*,*c)·Q*] axis.

To examine the structural interaction between CRP2 and actin filaments in solution, we analyzed the structural characters of rod like F-actin and CRP2 bound F-actin in solution by SAXS analysis. Fig 2A shows the SAXS data, $\bar{I(Q,c)}/c$, of F-actin with each CRP2 ratio. At the region around 0.1 Å^-1^ of the F-actin scattering data, the scattering valley and peak were slightly observed at 0.105 Å^-1^ (*Λ* = 60 Å, where *Λ* is the Bragg distance) and 0.11 Å^-1^ (*Λ* = 57 Å), respectively (Fig 2A). These valley and peak were characteristic of the SAXS profile for F-actin, indicating the actin periodical structure [26–28]. These characteristic profiles were also observed for CRP2 bound F-actin (Fig 2A). The decreased ratio of SAXS intensity for *Q* in each F-actin solution provides information on the shape of materials. The decreased ratio, *d*, between 0.01 < *Q* < 0.016 of each F-actin are shown in Table 1. From the scattering theory, *d = 1* indicates a rigid rod material, and *d = 2* indicates a flat disc material. The *d* of bare F-actin was approximately 1.4 indicating an intermediate object between rigid rod and flat disc shape. F-actin is a filamentous material having high flexibility [29,30]. Furthermore, low regional upward scattering of cylindrical objects can be ascribed to the flexibility of the cylindrical objects, to branching, or to large amounts of aggregation [31]. Thus, the *d* value of F-actin would be construed as ascribing to the flexibility of F-actin. With respect to CRP2, it was observed that as the CRP2 concentration increased, the *d* values increased. Thus, the macroscopic morphology of F-actin would change with the binding of CRP2. Since CRP2 is an actin binding protein, one possibility of macroscopic change is the formation of filament branching.

**Table 1:** Decreasing ratio (d) and cross sectional radius of gyration (Rc) of CRP2 bound F-actin.

F-actin: CRP2 ratio	*d*	*R*_*c*_ (Å)
1: 0	1.4 (0.01)	26.7 (0.24)
1: 1.5	2.2 (0.01)	26.9 (0.24)
1: 3.1	3.0 (0.00)	27.6 (0.23)
1: 4.6	3.4 (0.01)	25.2 (0.26)

Previous reports calculate cross sectional radius of gyration, *R*_*c*_, of F-actin using a modified Guinier plot for rod-like materials [26,32]. These reports fit the slope of the scattering intensity in the *Q* ≤ 0.063 A^-1^ regions. We then calculated the *R*_*c*_ of CRP2-bound F-actin. Fig 2B shows the modified Guinier plots for rod-like materials using the SAXS data (ln[*I(Q)·Q*] vs. *Q*^*2*^ plot) [20]. In the inner part of the Guinier plot *Q*^*2*^ < 0.002, there was upward scattering, and then we calculated the slope of the scattering in the 0.002 Å^-2^ ≤ *Q*^*2*^ ≤ 0.004 Å^-2^ region, which is the 0.045 Å^-1^ ≤ *Q* ≤ 0.063 Å^-1^ or 1.2 ≤ *Q·R*_*c*_ ≤ 1.7 region. The *R*_*c*_ of each F-actin solution is shown in Table 1. The *R*_*c*_ value of bare F-actin was determined to be 26.7 Å. This value is similar to previous reports of 25.7 Å [26]. The *R*_*c*_ values of F-actin were nearly constant with increasing CRP2 ratios (Table 1). Thus, it is assumed that CRP2 locates interface of the two adjacent actin subunits of cross sectional single F-actin; this location little affects the increase of *R*_*c*_ of F-actin (see Discussion).

### Cluster formation of CRP2-bound F-actin

**Fig 3:**
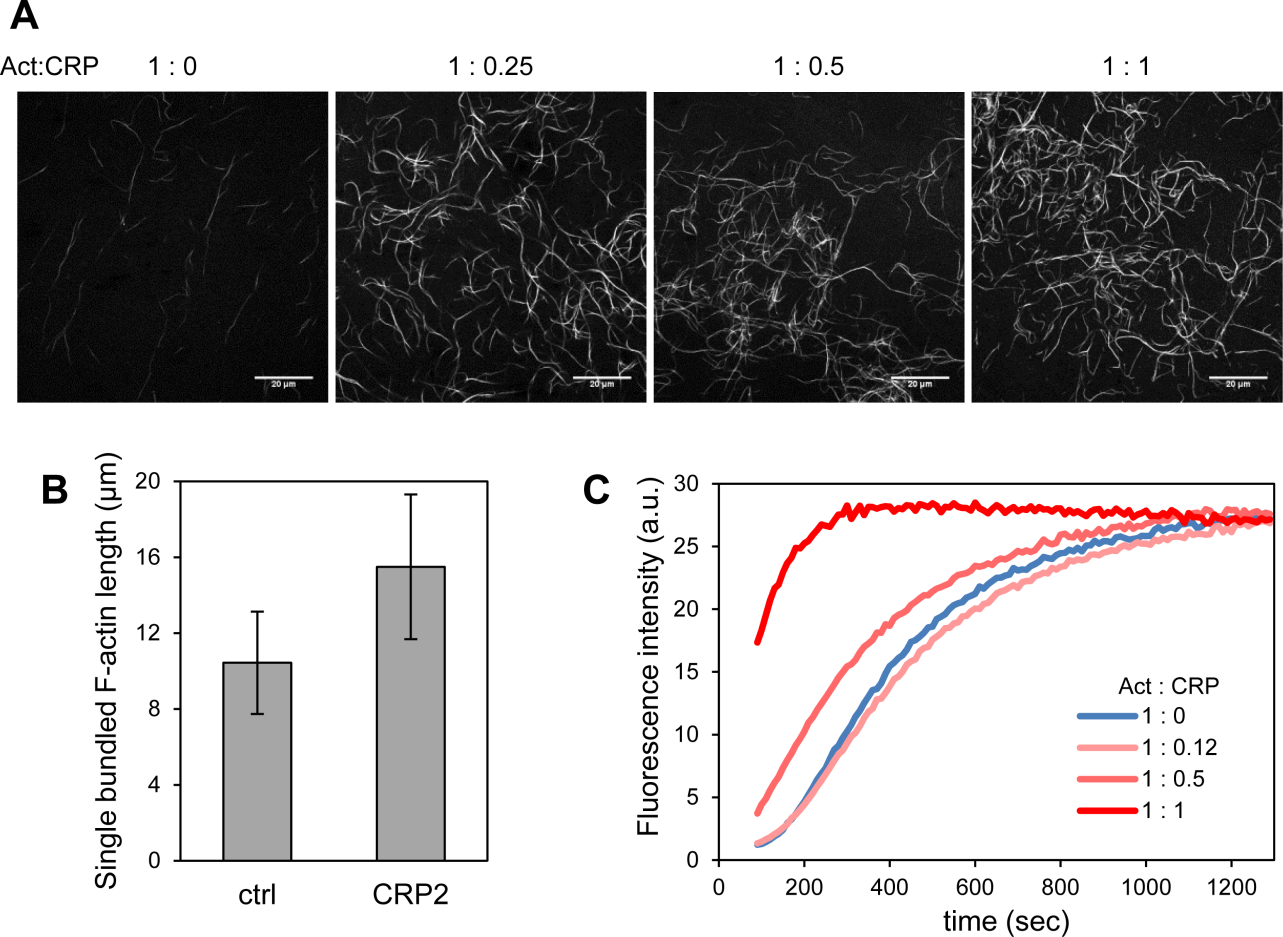
CRP2 accelerates actin filament formation and clustering. (A) Confocal laser scanning microscopy images of Alexa Fluor-labeled bare F-actin and CRP2-bound F-actin. Actin monomer (0.042 mg/mL) was incubated with or without CRP2 under filament-forming conditions for 4 h. The molar ratios of actin monomer:CRP2 were 1:0, 1:0.25, 1:0.5, and 1:1. (B) The lengths of single actin bundled filament with or without CRP2. Actin monomer (0.17 mg/mL) was incubated with or without 0.022 mg/mL CRP2 under filament-forming conditions for 4 h. The molar ratio of actin monomer:CRP2 was 1:0.25. The data shows the mean ± standard deviation (n = 40). *p* < 0.001. (C) Actin polymerization assay. Pyrene fluorescence of actin monomer (0.4 mg/mL) containing 10% pyrene-labeled was recorded under filament-forming conditions with or without CRP2. The molar ratios of actin monomer:CRP2 were 1:0, 1:0.12, 1:0.5, and 1:1.

Confocal laser scanning microscopy was used to observe F-actin structures and the formation of branches. We used Alexa Fluor 488-labeled actin monomers for polymerization. Fig 3A shows the fluorescence images of F-actin with or without CRP2. The filament clustering and complexity were clearly accelerated by the addition of CRP2 (Fig 3A). This held true even with the addition of small amounts of CRP2. We compared the length of each single bundled actin filament with or without CRP2 (Fig 3B). The length of each single bundled F-actin was clearly elongated by addition with CRP2. We furthermore examined actin polymerization kinetics using pyrene-actin assay; this method allows detection of filament formation by increase in pyrene fluorescence (Fig 3C). Actin polymerization was clearly accelerated by addition of CRP2 (Fig 3C). Recent study reports that CRP2 promotes F-actin bundling [19]. Thus, CRP2 accelerates actin polymerization, elongates filament length and promotes F-actin cluster formation.

**Fig 4:**
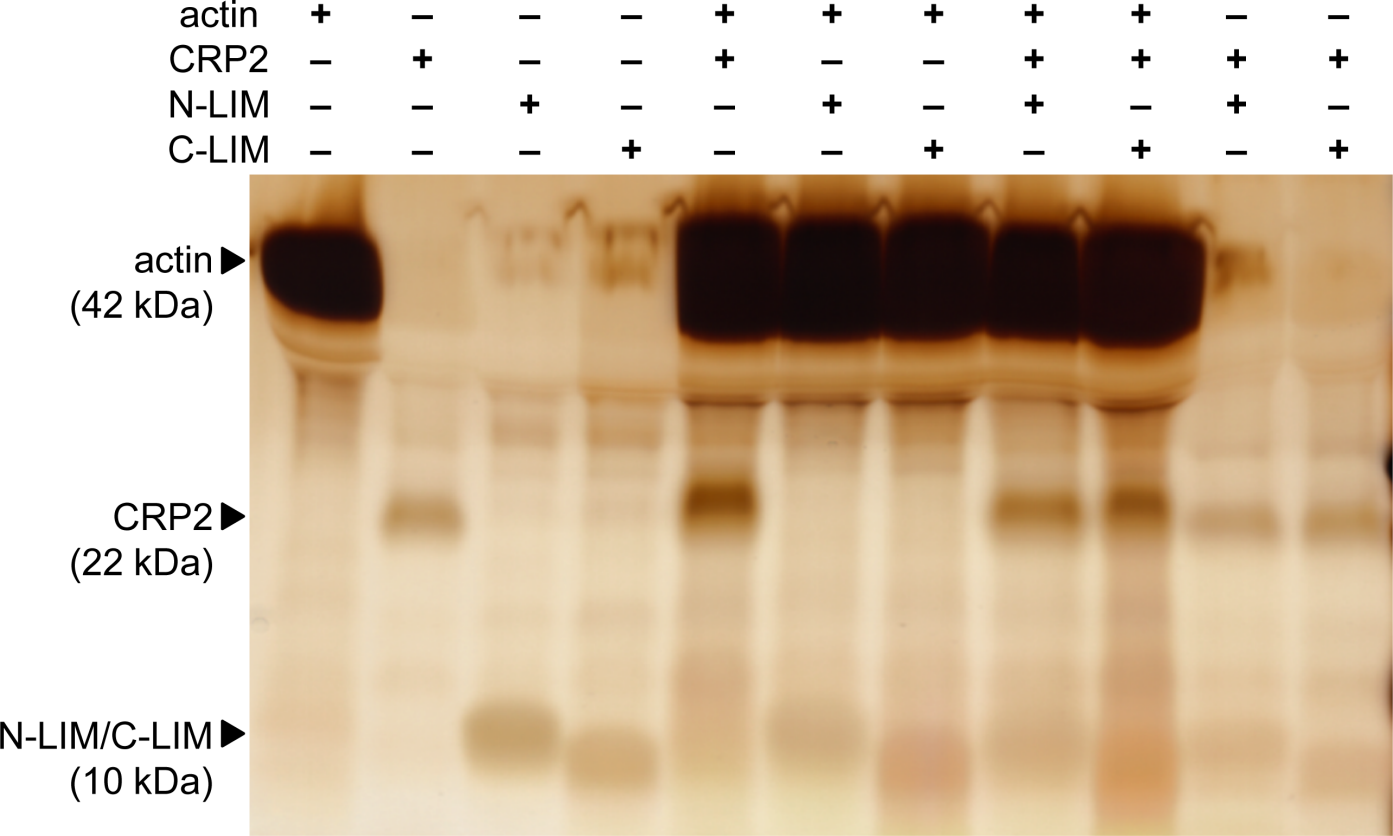
SDS-PAGE image of the pellets of low speed F-actin co-sedimentation assay. Actin monomer (0.17 mg/mL) was incubated with or without CRP2, N-terminal LIM domain and glycine-rich region (N-LIM), and C-terminal LIM domain and glycine-rich region (C-LIM) under filament-forming conditions with 8% PEG for 2 h. After that, formed F-actin was precipitated. The amount of CRP2 in the pellet clearly increased in the presence of F-actin, showing CRP2 directly bind to F-actin. The amount of CRP2 in the pellet together with F-actin decreased by addition with N-LIM. On the other hand, it was obscured whether the amount of N-LIM and C-LIM changed in the pellets.

We then examined the binding of CRP2 to bundled and clustered actin filaments by actin co-sedimentation assay. We used PEG for leading formation of higher order F-actin structure and then we precipitated the F-actin by low-speed (20,000 × g) centrifugation. G-actin protein was polymerized with or without full length, N-terminal LIM domain and glycine-rich region part (N-LIM), and C-terminal LIM domain and glycine-rich region part (C-LIM) of CRP2 proteins. It was considered that N-LIM or C-LIM of CRP2 influences the interaction between CRP2 and F-actin, because we previously revealed that N-LIM of CRP2 directly bound to F-actin [6]. Fig 4 shows the SDS-PAGE image of the resulting pellets of co-sedimentation assay. CRP2 accumulated in the pellet together with bundled F-actin (Fig 4). Furthermore, the amount of CRP2 decreased or unchanged under coexistence of N-LIM or C-LIM, respectively, in the pellet together with the F-actin (Fig 4). However, it was obscure whether only N-LIM or C-LIM accumulated in the pellet together with the F-actin; probably it was because molecular weights of these proteins were too small to compare the density of bands (Fig 4). Therefore, CRP2 directly bound to bundled F-actin, and N-LIM part inhibited the binding of CRP2 with bundled F-actin.

**Fig 5:**
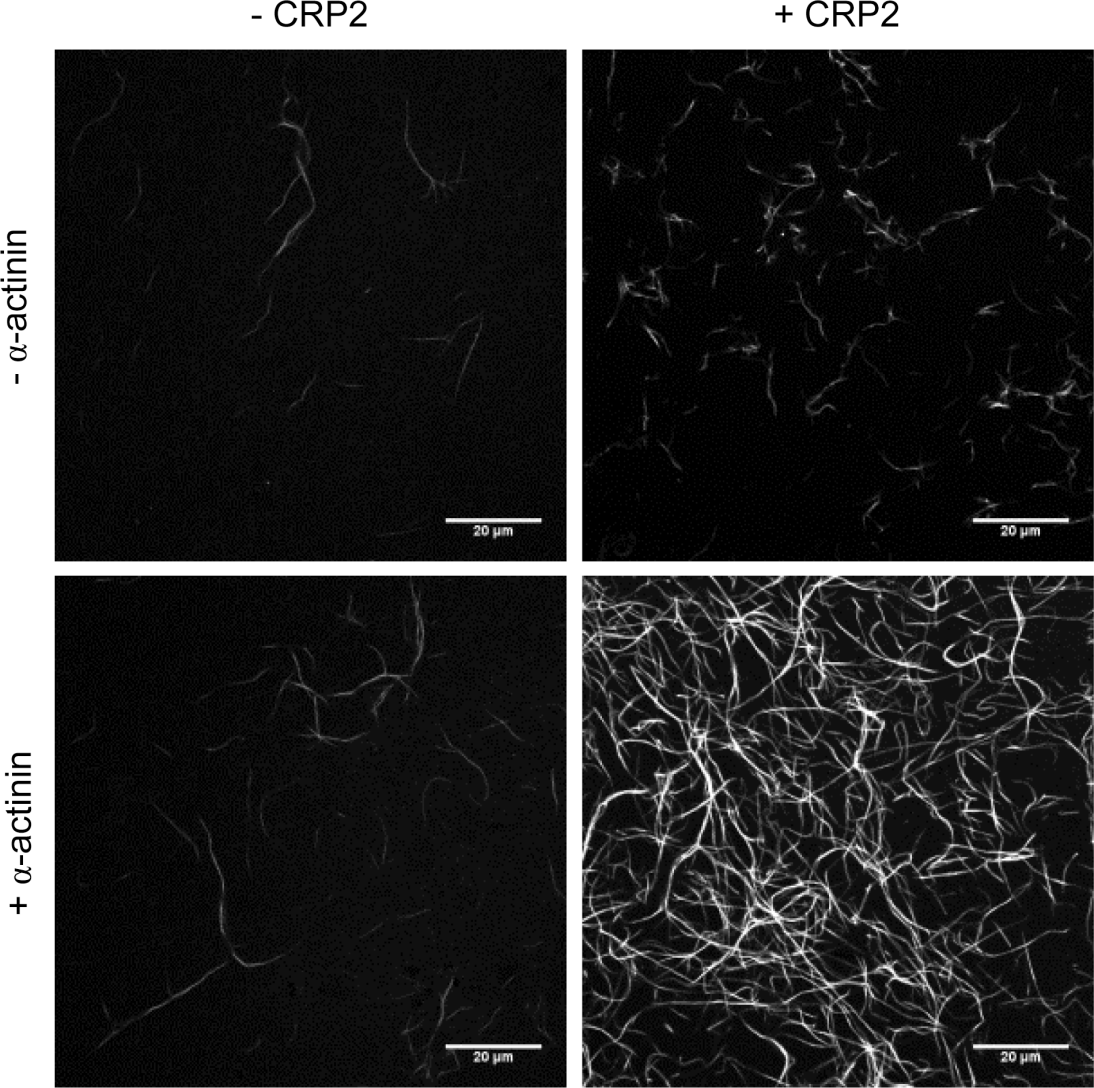
Confocal laser scanning microscopy images of Alexa Fluor-labeled F-actin with or without CRP2 and α-actinin. Actin monomer (0.042 mg/mL) was incubated with or without 0.01 mg/mL α-actinin and 0.022 mg/mL CRP2 under filament forming conditions for 2.5 h. The molar ratios of actin monomer:α-actinin:CRP2 was 1:0.1:1.

We finally examined the F-actin clustering activity of CRP2 in the presence of another actin crosslinking protein, α-actinin. Previous research has shown that CRP2 binds with α-actinin as well as actin filaments [6,10,14]. In our study, we first found that the formation and clustering activity of CRP2 was strongly augmented by the addition of α-actinin (Fig 5).

## Discussion

**Fig 6:**
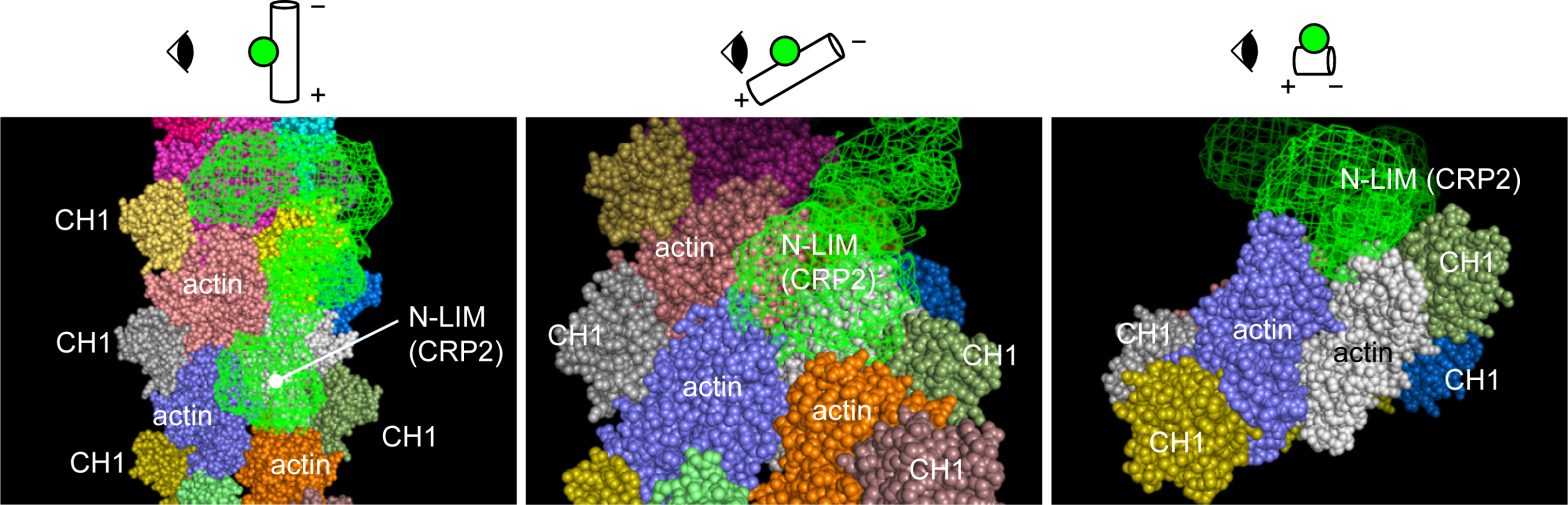
Structural model of interaction of CRP2 with F-actin. We used the molecular model of F-actin including CH1 domain of α-actinin (PDB ID: 3LUE) [34] and our CRP2 dummy model (S2 File). Each actin molecule and CH1 domain are shown as different colors, and CRP2 is shown as an object with green mesh. We speculate that N-terminal LIM domain of CRP2 locates at the interface of two adjacent actin subunits of cross sectional single F-actin and this position is closely to α-actinin binding position. The place of C-terminal LIM domain of CRP2 is probably independent of F-actin and α-actinin. The structural model was created by PyMOL software. Each viewpoint for the structural model is illustrated in each image. In the diagram, F-actin is shown as cylinder (+: plus end, -: minus end) and CRP2 is shown as green sphere.

CRP2 directly associates with F-actin through its N-terminal LIM domain and glycine-rich region [6,14]. CRP2 consists of two LIM domains and glycine-rich regions at both ends of a flexible linker region; the two LIM domains move freely and show independent space orientation [25]. The cross sectional radius of gyration *R*_*c*_ of bare and CRP2-bound F-actin were nearly identical with the value being approximately 26.7 Å. On the other hand, the *R*_*c*_ and *D*_*max*_ of CRP2 in solution were 15.9 Å and 118 Å, respectively. Thus, it is assumed that the N-terminal LIM domain of CRP2 is the contact point with interface of two adjacent actin subunits of cross sectional single F-actin, and the C-terminal LIM domain moves freely from the F-actin surface (Fig 6). The result of our co-sedimentation assay supports this idea; N-terminal LIM and glycine rich region inhibited the binding of full length of CRP2 with F-actin. On the other hand, CRP3, which is a CRP family protein, binds to actin filaments via its C-terminal LIM domain and self-associates via its N-terminal LIM domain, resulting in bundling of F-actin [33]. Previous reports [14,19] and our result clearly show that CRP2 also associates with F-actin directly and facilitates actin polymerization, crosslinking and clustering. Thus, although CRP3 self-associates via its N-terminal LIM domain [33], CRP2 could self-associate via the freely movable C-terminal LIM domain. However, our SAXS analysis did not show clear dimerization but aggregation of CRP2 in solution. Thus, we speculate that the self-association of CPP2 would be loose not rigorous. The loose self-associated CRP2 would work to crosslink the actin filaments and cause cluster formation.

The superstructure of F-actin is highly regulated by numerous actin-binding proteins, such as α-actinin, filamin, fascin, fimbrin, and scruin. These proteins form a variety of actin structures, ranging from meshworks to networks of thick bundles [35–38]. Of these actin-binding proteins, CRP2 associates with α-actinin [10,14], and probably N-terminal LIM domain of CRP2 interact with α-actinin [4]. α-actinin crosslinks F-actin with a disordered and loosely packed 33 nm lattice and forms branched 3D network structures [17]. Our data indicates that network formation of actin filaments caused by CRP2 is augmented by the coexistence of α-actinin. Interestingly, this augmentation effect could arise from even small amounts of α-actinin. α-actinin interacts with actin molecules with outside direction of F-actin (Fig 6) [34,39]. From our SAXS analysis, CRP2 will bind with the interface of the cross sectional two adjacent actin subunits of single F-actin. Thus, the interacting positions of CRP2 and α-actinin with F-actin will be different but closely, and then these molecules can act cooperatively to bundle and crosslink actin filaments (Fig 6).

During vascular smooth muscle development CRP2 expression increases and it translocates from the nucleus into the cytoplasm [4,40]. Particularly, CRP2 localizes to the actin cytoskeleton in smooth muscle cells [4,6]. Thus, it is of significant interest to understand the role that CRP2 might play while bound to the cytoskeleton in smooth muscle cells. In this study, we found that CRP2 accelerates actin polymerization and cluster formation of F-actin through its direct interaction with F-actin. Moreover, CRP2’s activity is cooperatively augmented by α-actinin. Future studies will address the influence of CRP2-bound F-actin in smooth muscle cells.

In conclusion, we examined the structure of CRP2 and CRP2 bound F-actin in solution by SAXS analysis. The CRP2 structure was relatively extended and consists of four tandem clusters. The cross sectional radius of gyration of F-actin was nearly constant even with the addition of CRP2. However, we found that CRP2 accelerated actin polymerization and complexity of F-actin structure by binding directly with F-actin. This effect of CRP2 was strongly augmented by addition of α-actinin. Our findings provide additional information of the function of CRP2 in actin filaments formation.

## Supporting information

S1 File The SAXS analyzed raw data file. (XLSX)

S2 File The PDB file of our presented dummy CRP2 model. (PDB)
